# Supplementary material for: Identification of an anti-CRISPR protein that inhibits the CRISPR-Cas type I-B system in Clostridioides difficile
Source: mSphere. 2023 Nov 27;8(6):e00401-23. doi: 10.1128/msphere.00401-23 (PMC10732046; doi:10.1128/msphere.00401-23)
Supplement: Supplemental material — Fig S1-S4; Tables S1-S7. [file msphere.00401-23-s0001.pdf]

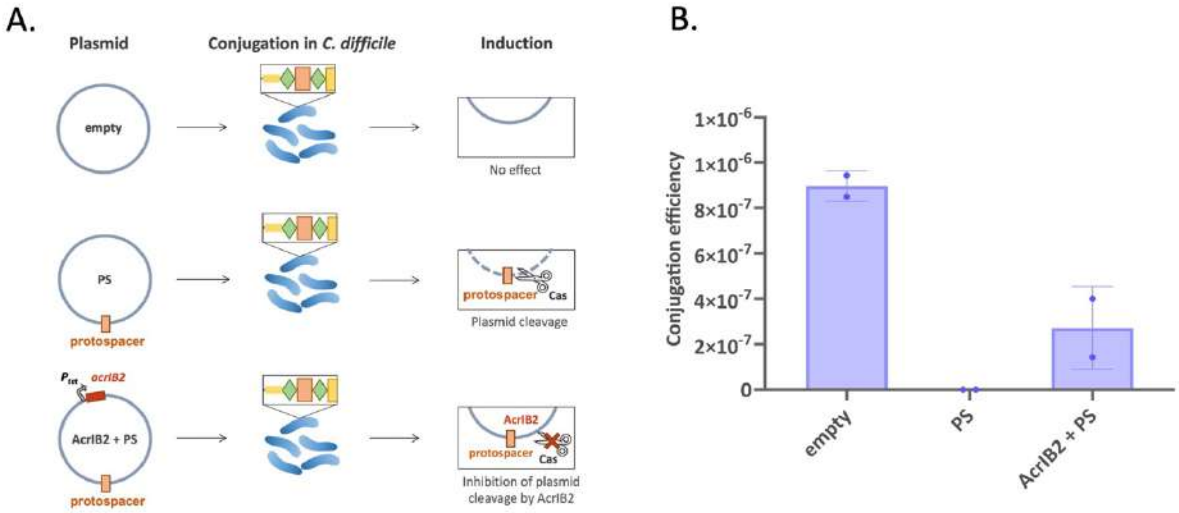

Supplementary Figure 1. Anti-CRISPR protein AcrIB2 partially inhibits CRISPR interference against a pRPF185Δgus plasmid carrying a protospacer.

(A) A plasmid loss strategy to reveal anti-CRISPR activity of plasmid-borne genes relies on a plasmid that carries a protospacer sequence (orange box) targeted by the crRNA spacer of the *C. difficile* 630Δerm CRISPR3 array. Green rhombi indicate CRISPR repeats, the blue rectangle indicates the spacer, the leader sequence is indicated in yellow. "PS" stands for protospacer plasmid, and "AcrIB2+PS" stands for protospacer plasmid with ATc-inducible *acrIB2* gene (red box). The control plasmid is referred to as "empty" vector.

(B) The conjugation efficiency of plasmids used for plasmid loss strategy is shown. Experiments were repeated two times, error bars represent the standard deviations between the means.

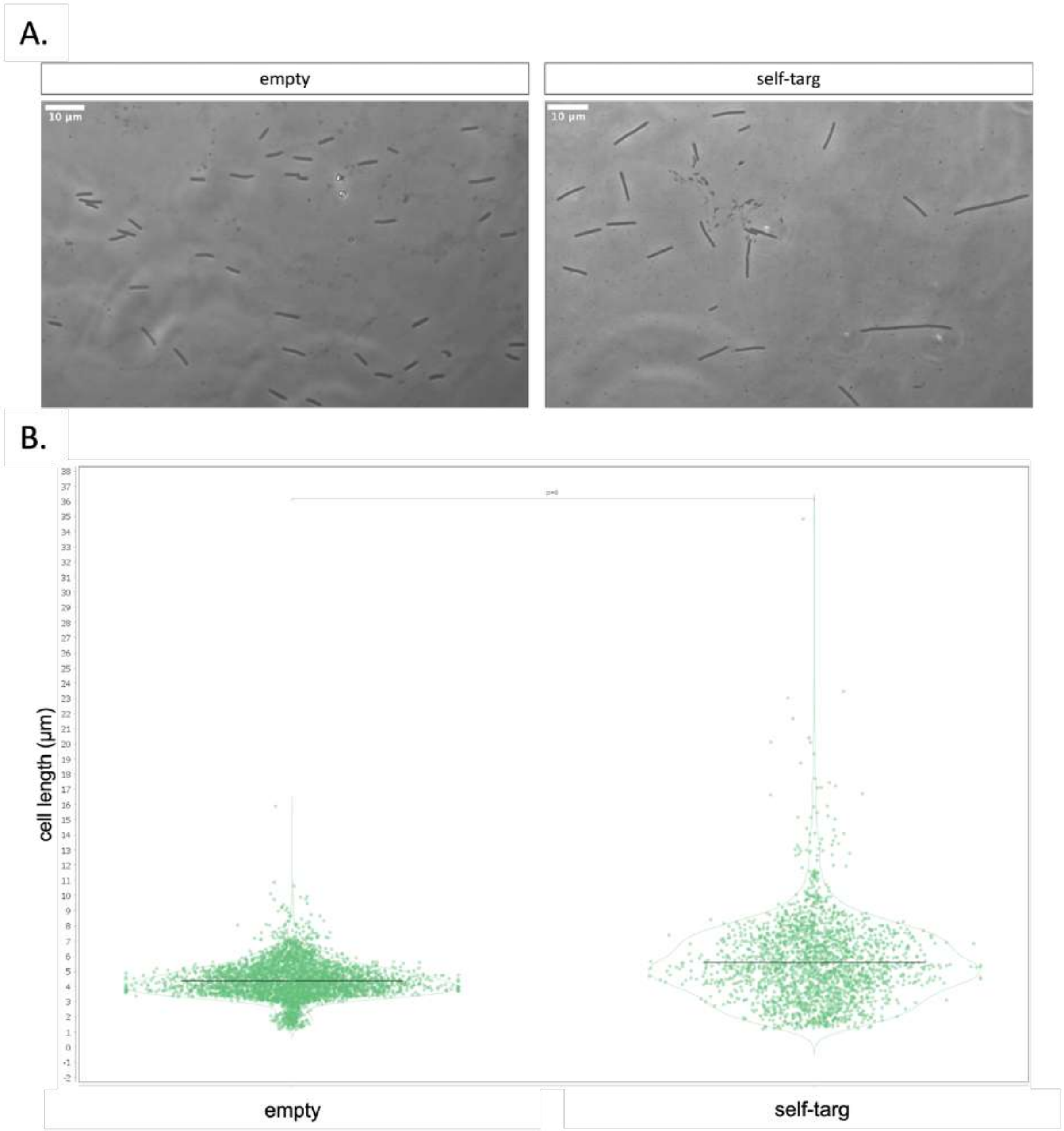

Supplementary Figure 2. SOS response in *C. difficile* cells

(A) The micrograph on the right shows a representative field of cells (x40 magnification) illustrating the effect of 3-hour induction of self-targeting plasmid-borne CRISPR array transcription on cell morphology. The micrograph on the left shows cells carrying the control plasmid without the mini CRISPR array grown in similar conditions.

(B) Violin plot illustrates the distribution of cell lengths (in micrometers, µm) in a population of cells bearing the control plasmid, absent of the mini CRISPR array, following a 3-hour ATc induction under similar environmental conditions. Cell length quantification was performed using Fiji software.

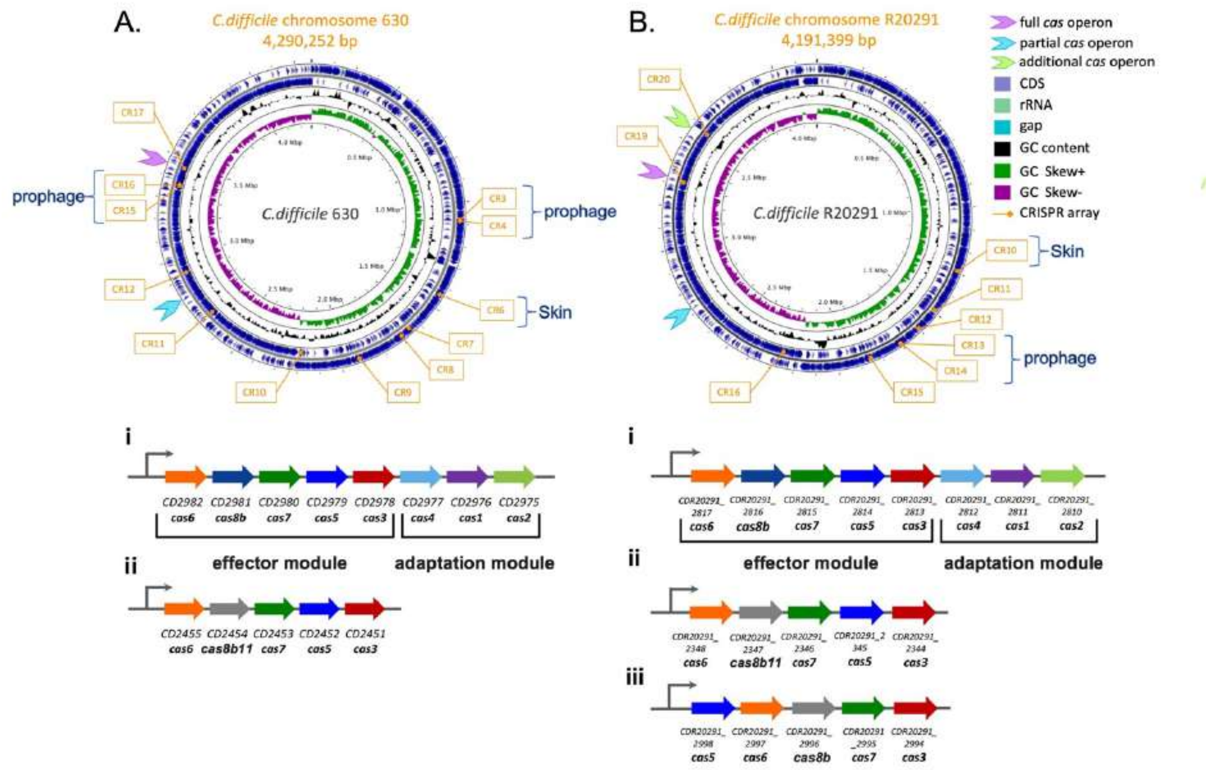

Supplementary Figure 3. Schematic view of CRISPR-Cas organization in the chromosome of *C. difficile* strains 630 (A) and R20291 (B). CRISPR arrays (CR) are numbered according to the CRISPRdb database (61). The locations of associated *cas* operons and prophage regions are indicated. The organization of the *cas* operons in strain 630 (A) and R20291 (B) are indicated with roman numerals, where i indicates full operon; ii – partial operon; iii – an additional partial operon. Functional modules are marked with brackets. Same colors are used to indicate homologous *cas* genes (27).

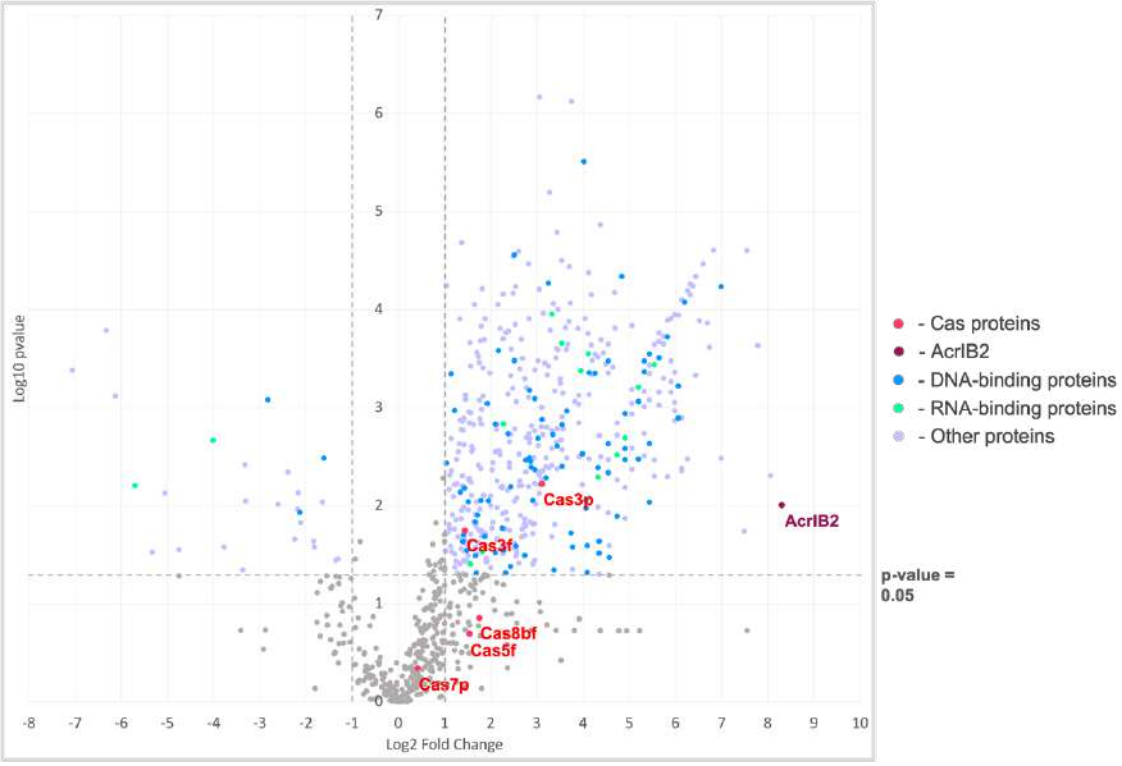

Supplementary Figure 4. Results of pull-down experiments for AcrIB2-N-Strep sample compared to the control.
Volcano plot from LC-MS/MS data demonstrates the magnitude (Log2 Fold change) and significance (Log10 p-value) for the AcrIB2-N-Strep sample compared to the control. The twofold change and p-value of 0.05 were used as the threshold cutoff. The horizontal dot-line shows the cut-off with the p-value equal to 0.05 [-log 10 (0.05) = 1.3]. The vertical lines show the cut-off equal to the two-fold change is 2 [log 2 (2) = 1] or 0.5 [log 2 (0.5) = -1]. Cas proteins are indicated in red. DNA-binding proteins are shown in blue, and RNA-binding proteins are colored in green. Violet dots correspond to all other proteins found in the LC-MS/MS data.

Supplementary Table 1. Mutations found in genomic DNA of escapers colonies of *C. difficile* 630Δ*erm* WT and Δfull *cas* operon mutant

Strain	Mutation	Position	Gene/Region	Translation
WT	Insertion of A	255688	CD630_01960 (Nickel carrier?)	Frameshift
	A>C	694997	Intergenic region	-
	G>C	1125053	Spacer1 of CRISPR3	-
	A>C	2069420	CD630_17860	N290H
	C>T	2663884	Leader seq of CRISPR11	-
	C>G	3397588	Spacer1 of CRISPR16	-
	T>G	4034625	CD630_34420	F63V
	Deletion (AAAAGTGCTAAAGCACGGCGCCAACGG ATAAATCCCGTTGCTT)	4150289	Intergenic region	-

Δfull *cas* operon	T>C	26911	Intergenic region	-
	G>T	26912	Intergenic region	-
	Insertion (+TAAATATATAGT)	2276149	PPS (*hfq* gene)	Modifies the 3' of the PPS sequence
	T>G	2316896	CD630_20061	STOP codon
	Deletion (AAAAGTGCTAAAGCACGGCGCCAACGGATAAATCCCGTTGCTT)	3152458	Intergenic region	-
	T>A	3397586	Spacer1 of CRISPR16	-
	A>G	3682242	Intergenic region	-
	T>C	3682243	Intergenic region	-
	G>A	3682337	Intergenic region	-
	T>C	3682342	Intergenic region	-
	A>C	3682343	Intergenic region	-
	A>G	3682346	Intergenic region	-
	T>C	3682347	Intergenic region	-
	T>C	3682349	Intergenic region	-
	Insertion of A	3972603	Intergenic region	-
	T>A	3999236	Intergenic region	-

Supplementary Table 2. The relative quantitative values of protein products of both *cas* operons in *C. difficile 630Δerm* found by liquid chromatography coupled to tandem mass spectrometry (LC-MS/MS).

**Cas protein**	**Molecular Weight**	**Number of unique peptides**			**Quantitative values (Total spectrum count)**			**Average**
		**W1**	**W2**	**W3**	**W1**	**W2**	**W3**	
**Partial cas operon**								
Cas6	29 kDa	4	4	6	4	4	7	5
Cas8b11	58 kDa	10	7	10	14	8	11	11
Cas7	39 kDa	11	12	13	19	21	22	21
Cas5	31 kDa	6	3	6	6	3	6	5
Cas3	94 kDa	15	14	14	20	18	20	19
**Full cas operon**								
Cas6	29 kDa	1		1	1		2	2
Cas8b	74 kDa	5	4	3	7	7	4	6

Cas7	35 kDa	6	6	7	6	8	8	7
Cas5	27 kDa	2	3	1	2	3	2	2
Cas3	90 kDa	1	1	1	1	1	1	1

Supplementary Table 3. Enriched DNA and RNA binding proteins from LC-MS/MS analysis of Strep-tagged AcrIB2 co-purification partners

Protein	Accession Number	Log10pvalue	logFC
**Enriched in Acr sample**			
Putative phage anti-repressor protein	CAJ69821.1	4.23392368	6.98489311
DNA polymerase I (POLI)	CAJ67980.1	4.07699065	6.19639721
Nuclease SbcCD subunit D	CAJ67883.1	2.89405772	6.05889369
Replicative DNA helicase, dnaC	CAJ70565.1	3.22179069	6.05889369
ATP-dependent nuclease subunit A (ATP-dependent helicase addA)	CAJ67882.1	3.1572409	5.9068906
DNA helicase	CAJ70042.1	3.72277902	5.82442844
Excinuclease ABC subunit B	CAJ70315.1	3.50728993	5.64385619
Regulator required for spore cortex synthesis	CAJ70420.1	3.44073347	5.54432052
Two-component response regulator	CAJ68999.1	2.03598631	5.43740531
Two-component sensor histidine kinase	CAJ67838.2	2.63491457	5.43740531
Transcriptional regulator, GntR family	CAJ67720.1	3.54911258	5.43740531
Transcriptional regulator, GntR family	CAJ69169.1	3.36657954	5.3219281
Transcriptional regulator, Rrf2 family (Transcriptional regulator of cysteine biosynthesis)	CAJ68134.1	3.47714002	5.3219281
Transcriptional regulator, DeoR family (Fatty acid and phospholipid biosynthesis regulator)	CAJ68031.1	2.47700872	5.19639721
Transcriptional regulator, MarR family	CAJ68155.1	3.06290705	5.19639721
Transcriptional regulator, RmlC-type	CAJ67972.1	3.06290706	5.19639721
putative rna-binding protein	CAJ68092.2	3.21029887	5.19639721
Transcriptional regulator, sigma-54-dependent	CAJ67223.2	2.47130795	4.9068906
Putative transcriptional repressor ccpn	CAJ69297.1	2.58364833	4.9068906
Putative RNA-binding protein	CAJ70385.2	2.69173013	4.9068906

Two-component sensor histidine kinase	CAJ70163.1	2.93892255	4.9068906
Stage 0 sporulation protein J, site-specific DNA-binding protein	CAJ70580.1	4.3400795	4.83650127
Putative trancriptional regulator	CAJ68250.3	1.89502776	4.73696559
Transcription antiterminator, PTS operon regulator	CAJ69215.1	2.51966547	4.73696559
Ribonuclease Z (RNase Z) (tRNase Z) (tRNA 3 endonuclease)	CAJ69426.1	2.51966547	4.73696559
Transcriptional regulator, RpiR family	CAJ67293.1	1.47372378	4.56478462
Transcriptional regulator, LysR family	CAJ69623.1	2.33772404	4.54432052
Putative recombinase Tn916-like, CTn7-Orf1	CAJ70270.1	2.33772404	4.54432052
DNA ligase (Polydeoxyribonucleotide synthase [NAD+])	CAJ70206.2	2.63431755	4.54432052
Transcriptional regulator, HTH-type	CAJ69049.1	2.63431755	4.54432052
ATP-dependent nuclease subunit B (ATP-dependent helicase addB), Superfamily 1 UvrD-family	CAJ67881.1	3.35520098	4.54432052
DNA polymerase III subunit beta	CAJ66816.2	3.47671331	4.54432052
Transcriptional regulator, sporulation initiation inhibitor, chromosome partitioning protein	CAJ70581.1	1.51773151	4.34577484
Transcriptional regulator, Penicillinase repressor	CAJ67299.1	1.51773151	4.34577484
Transcriptional regulator, TetR family	CAJ67411.1	1.6383849	4.34577484
Transcriptional regulator, Phage-type	CAJ69818.2	2.38787207	4.3219281
Putative integrase	CAJ68951.1	2.38787207	4.3219281
Transcription-repair coupling factor (TRCF ATP-dependent helicase mfd)	CAJ70405.1	3.35226791	4.26052755
Transcriptional regulator, TRAP family	CAJ70054.1	3.35667126	4.12928302
putative MiaB-like tRNA modifying enzyme	CAJ69335.1	3.55013636	4.11042399
Transposase IS200/IS605-like OrfA	CAJ67036.1	1.32049822	4.08746284
Transcriptional regulator, repressor-like	CAJ68553.1	1.5945306	4.08746284
Putative phage endodeoxyribonuclease RusA-like	CAJ69822.2	1.97670961	4.05889369

DNA mismatch repair protein	CAJ69030.1	1.97670961	4.05889369
Two-component response regulator, CheY family	CAJ70507.1	2.05743763	4.05889369
Two-component response regulator	CAJ68555.1	2.05743763	4.05889369
Excinuclease ABC subunit A	CAJ70314.1	5.50914301	4.01630181
Putative phage single-strand DNA-binding protein	CAJ69832.1	2.53038132	3.98489311
RNase Y	CAJ68187.2	3.37973388	3.94995932
Putative regulatory protease, U62 family	CAJ69755.1	1.58147828	3.7725895
Transcriptional regulator, Phage-type	CAJ70047.1	1.58147828	3.7725895
Transcription antiterminator, PTS operon regulator	CAJ66954.1	1.58147828	3.7725895
Transcriptional regulator, Phage-type	CAJ67740.1	1.72275663	3.73696559
Two-component sensor histidine kinase,sporulation-associated spo0A	CAJ68443.1	2.9666911	3.64385619
Transcriptional regulator, LysR family	CAJ69232.1	2.40234252	3.54432052
Helicase loader ATPase, dnaI	CAJ70562.1	2.82735682	3.54432052
Ribosomal RNA small subunit methyltransferase B (rRNA (cytosine-C(5)-)-methyltransferase)	CAJ69470.1	3.65752835	3.53765679
RNA polymerase sigma-54 factor	CAJ70073.1	2.60816581	3.43740531
Transcriptional regulator, sigma-54-dependent	CAJ68977.1	2.60816581	3.43740531
Transcriptional regulator, sigma-54-dependent	CAJ70083.2	1.345699	3.36923381
DNA repair protein RecO (Recombination protein O)	CAJ69321.1	1.345699	3.36923381
Nuclease SbcCD subunit C	CAJ67884.1	2.72766019	3.34557936
putative ATP-dependent RNA helicase	CAJ68991.1	3.95593674	3.3219281
Transcriptional regulator, CtsR family	CAJ66837.1	4.26978005	3.24792751
Flagellar number regulator FlhG	CAJ67087.1	2.28660351	3.19639721
DNA polymerase III, epsilon subunit	CAJ69279.1	2.28660351	3.19639721
Transcriptional regulator, GntR family	CAJ68245.1	2.88080357	3.11042399
Putative deoxyribonuclease	CAJ70443.1	2.68911167	3.02236781
DNA polymerase III DnaE subunit alpha	CAJ70299.1	2.36829751	2.95269429

Transcriptional regulator, Heat-inducible repressor HrcA	CAJ69350.1	3.09375514	2.95269429
Transcriptional regulator, RmlC-type	CAJ68509.1	2.0554654	2.9068906
PolIII (PolC) alpha subunit	CAJ68162.1	2.0554654	2.9068906
Two-component response regulator	CAJ68652.1	2.39396345	2.87446912
DNA binding protein	CAJ66831.1	2.45430724	2.86249648
Protein RecA (Recombinase A)	CAJ68186.1	3.17786772	2.83871909
Transcriptional regulator, Redox-sensing repressor Rex	CAJ66992.1	2.48954828	2.83650127
DNA mismatch repair protein MutS	CAJ68852.2	2.4673911	2.76004921
Transcriptional regulator, RpiR family	CAJ69937.1	1.49447717	2.73696559
Protein translocase subunit secA 2	CAJ69680.1	3.29638561	2.73696559
Transcriptional regulator, GntR family	CAJ70355.1	1.59124101	2.54432052
Transcriptional regulator, GntR family	CAJ67480.1	3.48230135	2.51457317
Transcriptional regulator, GntR family	CAJ70471.1	4.55783842	2.51245
DNA topoisomerase type IA	CAJ69289.1	2.19291717	2.4288433
Structural maintenance chromosome protein SMC	CAJ68105.1	1.38354683	2.42543267
Putative YaaT-like protein involved in sporulation or related to DNA replication	CAJ70452.1	2.73466432	2.36923381
DNA mismatch repair protein MutL	CAJ68851.1	1.31371811	2.3219281
Carbon storage regulator homolog CsrA	CAJ67055.1	2.83539685	2.27500705
DNA replication protein DnaD/DnaB	CAJ70561.1	1.5482005	2.27462238
RNA polymerase sigma factor SigA2 (sigma-43)	CAJ68363.1	1.770611	2.25153877
Transcriptional regulator, LacI family	CAJ67908.1	3.58226124	2.169925
Probable transcriptional regulatory protein	CAJ67629.1	1.52332543	2.09953567
RNA polymerase sigma factor SigA1 (sigma-43)	CAJ68320.1	2.83275048	2.09953567
Putative stage 0 sporulation protein,DNA-binding protein Spo0J-like	CAJ70582.1	2.05156972	1.94753258
DNA repair protein RecN (Recombination protein N)	CAJ68063.1	1.2838634	1.92961067
putative ferric-uptake regulator	CAJ67660.1	3.04438992	1.92471187
Transcriptional regulator, Fur family	CAJ68143.1	1.68859554	1.87126669

16S rRNA processing protein RimM	CAJ68111.1	1.53068248	1.81942775
DNA repair protein	CAJ66841.1	2.05413789	1.7884959
Transcriptional regulator, ArsR family	CAJ67392.1	1.90611119	1.71311885
DNA polymerase IV (Pol IV)	CAJ67732.1	1.31555568	1.68965988
Transcription antiterminator, PTS operon regulator, bglG4	CAJ70031.1	1.49158219	1.66481581
Transcriptional regulator, TPR family	CAJ70577.1	1.83584387	1.66343656
23S rRNA (uracil-5-)-methyltransferase RumA (23S rRNA(M-5-U1939)-methyltransferase)	CAJ70296.1	1.40701461	1.56071495
Transcription elongation protein	CAJ68164.1	2.03983206	1.50901365
Putative DNA recombination protein Tn1549-like,CTn5-Orf24	CAJ68739.1	1.54552253	1.4786536
Transcriptional regulator, SorC family	CAJ70072.1	2.17898164	1.4288433
Putative pyruvate, phosphate dikinase regulatory protein short=ppdk regulatory protein	CAJ69296.1	1.70440568	1.4150375
Transcription antitermination protein	CAJ66875.1	1.63600483	1.39231742
Transcription antiterminator, PTS operon regulator	CAJ69924.1	2.13839787	1.3439544
DNA helicase, UvrD/REP type	CAJ67150.1	2.9701866	1.21501289
DNA-directed RNA polymerase subunit beta	CAJ66881.1	3.34609298	1.13540912
Transcriptional regulator, GTP-sensing pleiotropic repressor codY	CAJ68131.1	2.43451271	1.04064198

Supplementary Table 4. List of clostridial phages and identified putative Acrs.

Phage	Putative *acr* gene name	Gene characteristics	Functionality	Reference for phage
AB012111.1				(Ohyama et al., 1995)
c-st				(Sakaguchi et al., 2005)
CDKM15				(Rashid et al., 2016)
CDKM9				(Rashid et al., 2016)
CDMH1	*phiCDHM1_gp29& phiCDHM11_gp30*	double orf		(Hargreaves, Kropinski, et al.,

				2014)
CDSH1	*CDHS1_28*	single orf		(Hargreaves, Kropinski, et al., 2014)
CD1801	*QVW56634.1*	single orf		(Whittle et al., 2022)
Clo-PEP-1				(Schoch et al., 2020)
CPS1				(Ha et al., 2019)
CPS2				(Ha et al., 2018)
CpV1				(Schoch et al., 2020)
D90210.1				(Kimura et al., 1990)
FN668943.1				(He et al., 2010)
JD032	*JD032_24*	single orf		(T. Li et al., 2020)
JD033	*JD033_49*	single orf		(Schoch et al., 2020)
phiCD211	*phiCD211_20148*	single orf		(Garneau et al., 2018)
HM_T				(Schüler et al., 2020)
HM2				(Schoch et al., 2020)
phiCDHM13	*phiCDHM13_gp26 & phiCDHM13_gp27, phiCDHM13_gp28 & phiCDHM13_gp29*	double orf	Non-functional	(Hargreaves, Kropinski, et al., 2014)
phi027				(Sekulovic & Fortier, 2015)
phi24R				(Morales et al., 2012)
phi34O				(Oakley et al., 2011)
phi3626				(Zimmer et al., 2002)
phi8074-B1				(Mayer et al., 2012)
phi9O				(Oakley et al., 2011)
phiC2				(Goh et al., 2005)
phiC2_isolate_584				(Goh et al., 2005)
phiC2_isolate_RW11				(Goh et al., 2005)
phiC2_isolate_RW2				(Goh et al., 2005)
phiCD111	*phiCD111_20028*	single orf		(Boudry et al., 2015; Sekulovic et

				al., 2014)
phiCD119				(Govind et al., 2006)
phiCD146	*phiCD146_20027*	single orf		(Boudry et al., 2015; Sekulovic et al., 2014)
phiCD24-1				(Boudry et al., 2015; Sekulovic et al., 2014)
phiCD24-2				(Boudry et al., 2015; Sekulovic et al., 2014)
phiCD27				(Mayer et al., 2008)
phiCD38-2	*phiCD38-2_gp27 (AcrIB2)*	single orf	Functional	(Sekulovic et al., 2011)
phiCD481-1	*phiCD48101_20029*	single orf		(Boudry et al., 2015; Sekulovic et al., 2014)
phiCD505				(Boudry et al., 2015; Sekulovic et al., 2014)
phiCD506	*phiCD506_20031*	single orf		(Boudry et al., 2015; Sekulovic et al., 2014)
phiCD52				(Boudry et al., 2015; Sekulovic et al., 2014)
phiCD630-1				(Soutourina et al., 2013)
phiCD630-2				(Soutourina et al., 2013)
phiCD6356				(Horgan et al., 2010)
phiCDHM11	*phiCDHM11_gp26 & phiCDHM11_gp27, phiCDHM11_gp28 & phiCDHM11_gp29*	double orf		(Hargreaves & Clokie, 2015)
phiCDHM14	*phiCDHM14_gp26 & phiCDHM14_gp27, phiCDHM14_gp28 & phiCDHM14_gp29*	double orf		(Hargreaves, Flores, et al., 2014)
phiCDHM19				(Hargreaves, Flores, et al., 2014)
phiCP13O				(Oakley et al., 2011)
phiCP26F				(Oakley et al., 2011)

phiCP7R				(Volozhantsev et al., 2012)
phiCPV4				(Volozhantsev et al., 2012)
phiCT19406A				(Cohen et al., 2017)
phiCT19406B				(Cohen et al., 2017)
phiCT19406C				(Cohen et al., 2017)
phiCT453A				(Cohen et al., 2017)
phiCT453B				(Cohen et al., 2017)
phiCT9441A				(Cohen et al., 2017)
phiCTC2A				(Cohen et al., 2017)
phiCTC2B				(Cohen et al., 2017)
phiCTP1				(Cohen et al., 2017)
phiMMP01				(Boudry et al., 2015; Meessen-Pinard et al., 2012)
phiMMP02				(Boudry et al., 2015; Meessen-Pinard et al., 2012)
phiMMP03				(Boudry et al., 2015; Meessen-Pinard et al., 2012)
phiMMP04				(Boudry et al., 2015; Meessen-Pinard et al., 2012)
phiS63				(Schoch et al., 2020)
phiSM101				(Myers et al., 2006)
phiZP2				(Volozhantsev et al., 2012)
susfortuna				(Pedersen et al., 2020)
vB_CpeS-CP51				(Gervasi et al., 2013)
39-O				(Seal et al., 2011)

Supplementary Table 5. Anti-CRISPR sequences used in this study, gBlocks, and regulatory elements.

Name	Sequence (5’-3’)

AcrIB2 (471 bp)	ATGAATAAACAAAAAGCTAGAAGATTTTTAAGAGTTATAGATATGAATATAGATAAAATAGAGGAA<br>GAAGCTATAAAAGCTTTTAAAGAAAGTTGTTTAATCAAAGAGACTAATAATATAAAAATTTATATCG<br>ATATACAAGGAAAAGTTGAAGCGATAGCAGTTCAAACTTGGGCTAAACTTTTAGGTGATGACAAA<br>GAAATTAATATTTTCACATTAAATCAAGCGCCAACTCATTTAAACGATATGCTTGGAGAAATTTGTTA<br>CGTAAACGATTATGAAGAATTTGAAAATTGGTGTGAAAATGAGTGGGAAAATTTGGATTGGGATA<br>GTTATAAAAAATTCAATAAAGAAAATTTCGAAGAAATTGCTGAAAGAAATATAGACGATAGCACAT<br>CAGTTTTTTTAGAAGAATTACAAAAAGGCATTGAAAGTTGTAAACAAGAATTGCAAAATGTAATTG<br>AAAATTAA
AcrIB2 (156 aa; 20 kDa)	MNKQKARRFLRVIDMNIDKIEEEAIKAFKESCLIKETNNIKIYIDIQGKVEAIAVQTWAKLLGDDKEINIF<br>TLNQAPTHLNDMLGEICYVNDYEEFENWCENEWENLDWDSYKKFNKENFEEIAERNIDDSTSVFLE<br>ELQKGIESCKQELQNVIEN
**gBlocks with regulatory elements**	
ϕCDHM13_gp26 (659bp; CDS 450bp)	AAGCACTGATTAGTACTATAACTCAATATAAGCATATCCCCTGGACTTCATGAAAAACTAAAAAAAA<br>TATTGACACTCTATCATTGATAGAGTATAATTAAAATAATAAACAAAGGGGGATATAAAAATGGAAA<br>AATTCATCAGACTTGATTACGATAAGGGCTTTAGAGGAAAAGAACATGTAAGTTCTGCAACTGGA<br>GATGGAGAACATTTTGAAGCAGGAATTAGTTGTTATAAAATAAGTAAAGAAAAATGTGTTGATGCT<br>ATAATAAATTTATGTGAATATTGGTTTGAATTTGCAGGTGAATGTCAATTCAAAGATTTCGATATAAA<br>TATTTTTGAAGGATGCTATGTAGGTGAGGGGGCTAGTTACGAAGATTTAGCCACTTGTGAGAAAC<br>ATTTATATACTGTAGATGGTTCTTTATTCAATGAAGTTTATGACTTATATTATATGCATGAAACATACTT<br>AGAAGAAAATGGAAATGTTGAAGAATTAGAAGAAAACTACAAAGATGAATATATAACAACAGAAG<br>AATTTGAAACTAAGATAAAAGAAATGTTTATAAAATATCTGTAGTAAATATTTATAGAGGCTATAAAT<br>AGCCTCTATTTTATGTGACTGTATCGTAACTAGAGAACCAAACGACGGAAAAAGCGAT
ϕCDHM13_gp27 (846bp; CDS 636bp)	AAGCACTGATTAGTACTATAACTCAATATAAGCATATCCCCTGGACTTCATGAAAAACTAAAAAAAA<br>TATTGACACTCTATCATTGATAGAGTATAATTAAAATATAATCAAAAGGAGGATATAAAAAATGAAAA<br>TTGGAGATAAATTTGAAAATCTTACAATACTAGATATAGAACAAAAAAATGGCAGGAAATATTGCTT<br>ATGTAAATGTAGTAATTGCAATAATGAAAAATGGATAAGGGCAGATAGTTTAAAAAGAATAAAAGC<br>ATGTGGATGCATGAAAAGTAACACACAGTTTAAACAAAATGATTTAACAGGCAAAAAGTTTGGCA<br>GATTAACAGCAATAAAGAACACTAATAAAAAAGCTAAAAGTGGTCACTATATTTGGGCTTGTAAGT<br>GTGATTGTGGAAATGAGATTAAGACAGCAGAAAACAATTTAACTACTGGTAGAACTAAATCATGTG<br>GATGCTTGAAAAAGGAATCTAATATAAAAAATGCAAAGATAGCGTTAAAAGTACATAAAGAAAAA<br>AATATTATTGATGATACAAATCTATCTATTATAAAAAAGACAGAAGCGTATTCTAATTCAAAAACTAA<br>AATTAGAGGCGTTTCTTGGAATAAAGAAAAAAGAAAATACTGTGCACAAATAGAATTTAAGAAAA<br>TGCATTATAATCTAGGATACTATGACAACATAAGAGAAGCAGAAGAAGCATACAAAAAAGCCAAA<br>GAAAAATTCTTAAAAGAAATAAACGGAAAAGTTTAATAAATATTTATAGAGGCTATAAATAGCCTCT<br>ATTTTATGTGACTGTATCGTAACTAGAGAACCAAACGACGGAAAAAGCGAT

ϕCDHM13_gp28 (525bp; CDS 315bp)	AAGCACTGATTAGTACTATAACTCAATATAAGCATATCCCCTGGACTTCATGAAAAACTAAAAAAAATATTGACACTCTATCATTGATAGAGTATAATTAAAATATAATCAAAAGGAGGATATAAAAAATGAATAAACAAAAAGCTAGAAGATTTTTAAGAGTTATAGATATGAATATAGATAAAATAGAAGAAGAAGCTATAAAAGCTTTTAAAGAAAGTTGTTTAATCAAAGAGACTAATGATATAAAAATTTATATCGATATACAAGGAAAAGTTGAAGCGATAGCAGTTCAAACTTGGGCTAAACTTTTAGATGATGACAAAGAAATTAATATTTTCACATTAAATCAAGCACCAACTCATTTAGACGATATGTTTGGAGAAATTTACCATGTAAATGATTATGAAGAGTTCGAAAAATGGTGTGAAAAAGGAAGATAATAAATATTTATAGAGGCTATAAATAGCCTCTATTTTATGTGACTGTATCGTAACTAGAGAACCAAACGACGGAAAAAGCGAT
ϕCDHM13_gp29 (384bp; CDS 174bp)	AAGCACTGATTAGTACTATAACTCAATATAAGCATATCCCCTGGACTTCATGAAAAACTAAAAAAAATATTGACACTCTATCATTGATAGAGTATAATTAAAATATAATCAAAAGGAGGATATAAAAAGTGAAAAAGGAAGATAAAAGTTTGAATTGGGATAGTTATGAAAAATTCAATAAAGAAAATTTCGAAGAAATCGCTGAAAGAAATATAGATGATAGCTCATCAGCTTTTTTAGAAAAATTACGTGAAGGCATTGAAAATTGTAAACAAGAATTACAAAATATAGTTGAAAATTAATAAATATTTATAGAGGCTATAAATAGCCTCTATTTTATGTGACTGTATCGTAACTAGAGAACCAAACGACGGAAAAAGCGAT
**Regulatory elements in gBlocks**	
Left homology arm	AAGCACTGATTAGTACTATAACTCAATATAAGCATATCCC
P_{tet} promoter	CTGGACTTCATGAAAAACTAAAAAAAATATTGACACTCTATCATTGATAGAGTATAATTAAAATA
RBS	TAATCAAAAGGAGGATATAAAAA
Terminator	TAAATATTTATAGAGGCTATAAATAGCCTCTATTTTATGTGA
Right homology arm	CTGTATCGTAACTAGAGAACCAAACGACGGAAAAAGCGAT

Supplementary Table 6. Bacterial strains and plasmids used in this study.

Strain	Genotype	Source
	E. coli	
NEB-10 beta	*Δ(ara-leu) 7697 araD139 fhuA ΔlacX74 galK16 galE15 e14- ϕ80dlacZΔM15 recA1 relA1 endA1 nupG rpsL (StrR) rph spoT1 Δ(mrrhsdRMS-mcrBC)*	New England Biolabs

HB101 (RP4)	*supE44 aa14 galK2 lacY1 Δ(gpt-proA) 62 rpsL20* (StrR)*xyl-5 mtl-1 recA13* Δ*(mcrC-mrr) hsdSB (rBmB-)* RP4 (Tra+ IncP ApR KmR TcR)	Laboratory stock
	C. difficile	
630Δ*erm*	Sequenced reference strain Δ*ermB*	Laboratory stock (Hussain et al., 2005)
R20291	PCR-ribotype 027 epidemic strain	Laboratory stock
CD125	R20291 carrying ϕCD38-2 prophage	Sekulovic and Fortier, 2015
CDIP741	630Δ*erm* Δ*CD2975-2982* (full *cas* operon)	Laboratory stock (Maikova et al. 2018)
CNRS_CD270	630Δ*erm* Δ*CD630_2455* (partial *cas* operon)	This work
CNRS_CD564	630Δ*erm* Δ*CD2975-2982* (full *cas* operon) and Δ*CD630_2455* (partial *cas* operon) double mutant	This work
	**Plasmids**	
pDIA6103	pRPF185Δ*gus* vector derivative	(Fagan and Fairweather, 2011; Soutourina et al., 2013)
pDIA6435	pRPF185Δ*gus* with the 5' CCA-PAM protospacer, corresponding to the spacer1 from 630Δ*erm* CRISPR3 array	Laboratory stock (Maikova et al. 2021)
pDIA6555	pDIA6103 carrying the *hfq* gene targeting CRISPR mini-array with the partial leader sequence under the control of P*tet* promoter	Laboratory stock (Maikova et al. 2019)
p074	pDIA6555 + *acrIB2*	This work
p173	pDIA6555 + *acrIB2*- N-Strep tag	This work
	**Editing Plasmids**	
p028	pMSR14 Δ*cas* CD630/2451-2455 partial operon	Laboratory stock (Maikova et al. 2018)

p233	pMSR14 + Δ*cas* CD630/2975-82 full operon	This work

Supplementary Table 7. List of primers used in this study.

Name	Sequence	Purpose
JP403	CTGTATCGTAACTAGAGAACCAAAC	Linearization of pRPF185-derived plasmids
JP404	GGGATATGCTTATATTGAGTTATAGTAC	
AS005	agtactataactcaatataagcatatcccTTGACTTAGATATTCATATAGATTATAATATAAAAATGGAGGATATAAAAAATGAATAAAC	Cloning the *AcrIB2 (gp27)* into pDIA6103
AS006	ccgtcgtttggttctctagttacgatacagCATAATAACTAAACATTAAAAAACAGTGT	
AS007	taattaaaatataatcaaaaggaggatataaaaaatgaatAAACAAAAAG	
AS008	ATTCATTTTTTATATCCTCCTTTTGATTA	
PM_3	TGGCTCCACGCGCTCATTTTTTATATCCTCCTTTTG	STREP insertion
PM_4	CCCGCAGTTCGAAAAAAATAAACAAAAAGCTAGAAG	
PM_5	GGCTCCACGCGCTATTTTCAATTACATTTTG	
PM_6	ACCCGCAGTTCGAAAAATAAGTAGAAGAGTATTATTATTAATG	
PM_7	GTGGCTCCACATTTTTTATATCCTCCTTTTG	
PM_8	CCGCAGTTCGAAAAAAATAAACAAAAAGCTAGAAG	
PM_9	GGGTGGCTCCAATTTTCAATTACATTTTGCAATTCTTG	
PM_10	GCAGTTCGAAAAATAAGTAGAAGAGTATTATTATTAATG	
PM_11	ATAAGCATATCCCCTGGACTTC	Check-up of STREP insertion
PM_12	TTTTTCGAACTGCGGGTGGCTCCA	
PM_13	TGGAGCCACCCGCAGTTCGAAAAA	
PM_14	GAATACACTGTTTTTATTTCCGCAT	
PM_42	GTATAAGTCTCCTCTCAATCATC	*hfq* interference screening
PM_43	ATGCAAAACGTCAGATAACATGG	
PM_44	CTTTCCAGGTTGTATAGTTG	
PM_45	GTTCAATTTAATCTTGGGAGGGTAC	
PM_69	cggtgtttttgttaccctaagtttGAGATAGAAATTATTTTTATTATACGTTTTTTTG	*cas* delition *CD630_2982* full operon
PM_70	gttaatatattaattgagagaggtgatattatgTAGGTTTGCAGTGAGCGATATTTATGC	
PM_71	gcataaatatcgctcactgcaaacctaCATAATATCACCTCTCTCAATTAATATATTAAC	

PM_72	ggtcatgagattatcaaaaaggagtttGATAGTGAAGTTTCAATTCTAGATATGATA	
